# The effect of silver nanoparticles on composite shear bond strength to dentin with different adhesion protocols

**DOI:** 10.1590/1678-7757-2016-0391

**Published:** 2017

**Authors:** KOOHPEIMA Fatemeh, MOKHTARI Mohammad Javad, KHALAFI Samaneh

**Affiliations:** 1 Shiraz University of Medical Sciences, School of Dentistry, Biomaterial Research Center, Department of Operative Dentistry, Shiraz, Iran.; 2 Islamic Azad University, Zarghan Branch, Young Researchers and Elite Club, Zarghan, Iran.

**Keywords:** Silver, Nanoparticles, Shear strength, Dentin-bonding agents

## Abstract

In Dentistry, restorative materials and oral bacteria are believed to be responsible for restoration failure. To make long-lasting restorations, antibacterial agents should be made. Inorganic nanoparticles and their nano composites are applied as good antibacterial agents.

**Objective:**

The purpose of this study was to investigate the effect of silver nanoparticles on composite shear bond strength using one etch and rinse and one self-etch adhesive systems.

**Material and Methods:**

Silver nanoparticles were prepared. Transmission electron microscope and X-ray diffraction were used to characterize the structure of the particles. Nanoparticles were applied on exposed dentin and then different adhesives and composites were applied. All samples were tested by universal testing machine and shear bond strength was assesed.

**Results:**

Particles with average diameter of about 20 nm and spherical shape were found. Moreover, it was shown that pretreatment by silver nanoparticles enhanced shear bond strength in both etch and rinse, and in self-etch adhesive systems (p≤0.05).

**Conclusions:**

Considering the positive antibacterial effects of silver nanoparticles, using them is recommended in restorative dentistry. It seems that silver nanoparticles could have positive effects on bond strength of both etch-and-rinse and self-etch adhesive systems. The best results of silver nanoparticles have been achieved with Adper Single Bond and before acid etching.

## Introduction

Silver nanoparticles (NPs) have called the researcher’s attention because of their unique properties, for example, their optical, antimicrobial, and electrical properties^15^. The antimicrobial, antifungal and antiviral action of silver or silver compounds depends on the level of released bioactive silver ions (Ag^+^) and its availability to interact with bacterial or fungal cell membranes^14^. Silver NPs have the potential to serve as a bactericidal agent because of the inherent antimicrobial influences of silver ion. NPs are insoluble particles that are smaller than 100 nm in diameter^25^. They benefit smaller particles that demonstrated stronger antibacterial activity compared to larger particles due to their higher surface area and, thus, enhanced interaction with organic and inorganic molecules. However, many properties of metal NPs are still unknown^27^. The potency of the antibacterial effects corresponds to the nanoparticle size. Smaller particles have more antibacterial properties because of the equivalent silver mass content^23^. Silver NPs adhere and penetrate the bacterial cell wall, thereby causing structural changes in the cell membrane, such asin cell membrane permeability and cell apoptosis. There is a formation of pits on the cell surface, and there is accumulation of NPs on the cell surface^9^. Silver NPs may have potential toxicities at some concentrations and can produce many health problems if used inappropriately. Metal NPs have been recently applied in Dentistry because of their bactericidal and bacteriostatic effects^11^.

Caries preventive measures with the purpose of reducing demineralization should be independent of the patient’s compliance^5^. These preventive measures consist of antimicrobial bonding agents, antibacterial mouth rinses, and remineralizing agents adjacent to oral appliances. It has been demonstrated that the presence of silver in dental restorative materials is effective against caries producing bacteria, such as streptococci and lactobacillus^19^. It can also be effective as an antibacterial additive to dental restorations. We do not know exactly whether the incorporation of silver NPs would interfere with bond strength of dental restorative materials^16,19^. Until now, only few studies have determined the effect of silver NPs on composite shear bond strength (SBS). Adhesion mechanism is based on the penetration of resin molecules into enamel and dentin^22^. Bond strength of dental composites to dentin is one of the main criteria in clinical durability of composite restoration. Various bonding systems have been introduced in order to fulfill a reliable bond to tooth structure based on two main methods: the etch-and-rinse and the self-etching adhesive systems^7^. Smear layer removal and formation of collagen fibril layer by means of acid conditioner to form hybrid layer are the main adhesion mechanisms in etch-and-rinse systems^3^. The self-etching adhesive system is subdivided into two groups: two-step and one-step self-etching. Self-etching adhesive systems employ an acidic monomer as conditioner. In self-etch adhesives, acidic functional monomers react to the mineral content of tooth surface^24^. Self-etch adhesives are less time-consuming and technique-sensitive. Etch-and-rinse adhesive systems are still considered as a golden standard among bonding systems. However, dentists have a tendency to use adhesive systems with a simplified application procedure^12^.

Therefore, in this study we examined whether an additional pretreatment with silver NPs provides any supplementary effect on bond strength of self-etch commercial adhesive (Clearfil SE Bond) and etch-and-rinse (Adper Single Bond) using standard SBS methodology.

## Material and methods

### Preparation and characterization of silver NPs

Portions of 1 g/L AgNO_3_ with sodium dodecyl sulfate (SDS) (Merck) were mixed in an aqueous solution under a N2 atmosphere. SDS molecules were applied to disperse the silver NPs and also to stabilize the formation of shaped silver NPs. Deposition was carried out at different reaction times. Oxygen was removed from water by nitrogen bubbling and the electrolyte was combined under a nitrogen atmosphere. SDS was added at the 40 g/L level to avoid aggregation. The products were washed with distilled water and collected by centrifugation at 15,000 rpm for 10 min (Hettich Universal 320, Tuttlingen, Germany). A transmission electron microscope (TEM) (JEM-1011, JEOL, Japan) was used to determine size, shape, and size distribution of silver NPs. Silver NPs were prepared by placing a drop of working solution on a TEM grid and dryed for TEM analysis. X-ray diffraction (XRD) was used as a secondary or complementary technique to determine particle size by analyzing diffraction peaks. XRD pattern of silver NPs was obtained by a DX-1000 X-ray powder diffractometer (DX-1000X, Dandong Fangyuan, China)^17^.

### Specimen preparation

Ninety extracted, noncarious human premolar teeth were cleaned and stored in 0.1% thymol solution for one week. The teeth were prepared using a diamond bur (4138 KG Sorensen, Barueri, Brazil) and a high-speed handpiece under water coolant up to the nearmost half of the DEJ to the pulp, which is the proposed location for measuring bond strength of composites, so that no pulp exposure occur in the preparation site. Then the exposed superficial dentin surface was polished using silicon carbide paper (600 grit) under water coolant to standardize the smear layer. The teeth were rinsed with distilled water to remove any debris and then mounted in acrylic resin (2×3×5 cm) and randomly divided into six groups (n=15).

The specimens’ preparation was based on the type of employed adhesive system and tested silver NPs as follows:

Group 1: Acid etching + Adper single Bond + Composite Z250

Group 2: Silver NPs + Acid etching + Adper single Bond + Composite Z250

Group 3: Acid etching + Silver NPs + Adper single Bond + Composite Z250

Group 4: Clearfil SE Bond Primer + Clearfil SE Bond + Composite Z250

Group 5: Silver NPs + Clearfil SE Bond Primer + Clearfil SE Bond + Composite Z250

Group 6: Clearfil SE Bond Primer + Silver NPs + Clearfil SE Bond + Composite Z250

**Figure 1:**
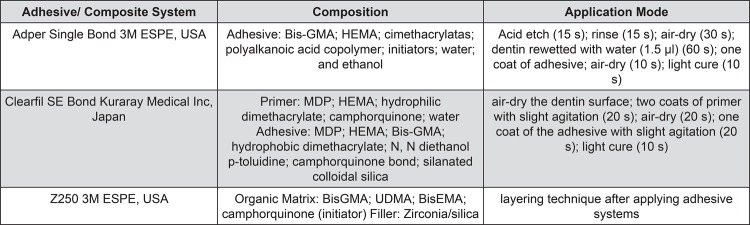
Adhesive and composite systems, composition, and mode of application

In all groups tested the application of silver NPs were followed by a 60-second rinse with water. Adhesive systems were applied following the manufacturer’s instructions (Figure 1). Subsequent to the application of adhesive on dentin surface, a resin composite block (3M ESPE Filtek Z250, St Paul Minnesota, USA) was built up over the bonded dentin with the aid of a Teflon mold (2 mm height×5 mm diameter) at room temperature, followed by light-curing for 40 seconds in vertical position on composite surface (600 mW/cm^2^)^4,26^.

### SBS measurement

Specimens were stored in distilled water at 37°C for one week and then they were tested in shear mode using a rod attached to the universal testing machine (Zwick/Roell Z020, Stuttgart, Germany) at a cross-head speed of 0.5 mm/min until the bond fractured and the shear force were evaluated. The force was measured in Newtons (N). SBS values were calculated by converting Newtons into megapascals (MPa)^4^.

### Statistical analysis

Statistical analysis was performed using SPSS 18.0.0 software (SPSS Inc, Chicago, USA). The statistically significant difference among groups was determined using analysis of variance (ANOVA) and Tukey test with the level of significance at p=0.05.

## Results

### TEM and XRD analysis

**Figure 2:**
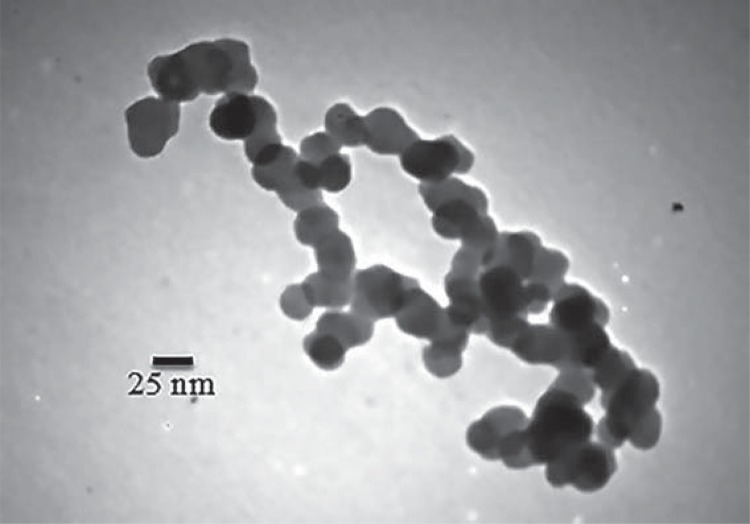
Transmission electron microscope (TEM) image of spherical silver nanoparticles (NPs) and their particle size distributions. TEM image shows silver particles with average diameter of about 20 nm and spherical shape

The size and morphology of particles were assessed using a TEM (Figure 2). TEM image shows silver particles with average diameter of about 20 nm and with spherical shape.

**Figure 3:**
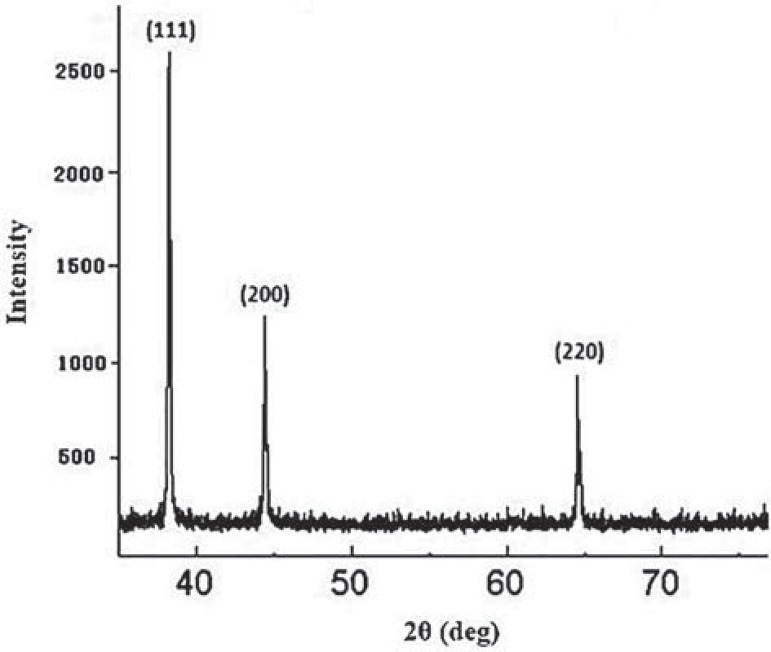
X-ray diffraction (XRD) pattern of silver nanoparticles (NPs). Peaks are assigned to diffraction from the (111), (200) and (220) planes of silver. Peaks of XRD pattern can be indexed as a face centered cubic (fcc) structure (JCPDS, file no. 4-0783). XRD pattern shows the presence of the diffraction peaks corresponding to the (111), (200), and (220) planes

A typical XRD pattern of the as-prepared silver NPs is shown in Figure 3. All peaks in the XRD pattern can be indexed as a face centered cubic (fcc) structure (JCPDS, file no. 4- 0783). XRD pattern shows the presence of diffraction peaks corresponding to (111), (200), and (220) planes. Mean particle size determined using the Scherrer method on the main (111) diffraction peak of the X-ray diffraction pattern is 20±2 nm, agreeing with the size of isolated NPs observed in TEM.

### SBS measurement

**Figure 4:**
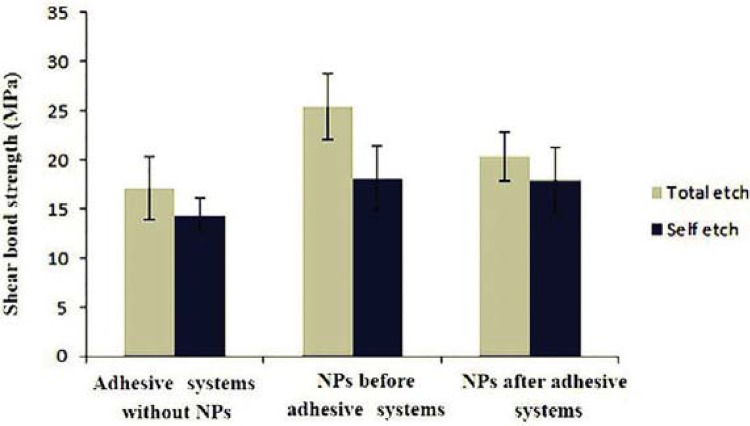
Means and standard deviations of shear bond strength (SBS) for the groups

Table 1 and Figure 4 demonstrated mean SBS values and standard deviation of the two adhesive systems at the three tested conditions. Maximum and minimum SBS in the presence of silver NPs were observed in group 2 (25.42±3.36, p=0.000) and group 6 (17.95±3.31, p=0.05), respectively.

**Table 1:** Descriptive statistics of the groups and comparison of shear bond strength (SBS) values

Column 1	Column 2	Column 3	Column 4	Column 5
Group	Code	n	Mean±SD [MPa]	Significance *P≤0.05, **P≤0.01, *** P≤0.001
1	A	15	17.10±3.18	A,B***
2	B	15	25.42±3.36	B,A*** B,C**
3	C	15	20.34±2.50	C,B**
4	D	15	14.34±1.75	D,E* D,F*
5	E	15	18.08±3.28	E,D*
6	F	15	17.95±3.31	F,D*

## Discussion

Nanotechnology is making advances in many fields, specially in Dentistry. In this technology, the specific material is converted into nanometric sizes for showing new properties^21^. Antibacterial properties of silver NPs and their compounds have been used in various medical and recently dental branches^16^. Due to the potential of microorganisms to stick to composite resins and adhesives compared to other restorative materials, the present study made an attempt to evaluate the effect of silver NPs on composite SBS with different adhesion protocols. In this study, etch-and-rinse adhesive with the application of silver NPs before acid etching showed better results. The use of silver NPs also had no adverse effect on bond strength in other groups.

Silver is a safe bactericidal metal because it is biocompatible to animal cells but is very toxic to bacteria^16^. Smaller silver NPs (<100 nm) can interact very closely with microbes. They provide a larger surface area for antimicrobial activity because of greater ratio of surface-to-volume than larger particles^1^. A variety of preparation methods have been demonstrated for the synthesis of silver NPs; some of them are laser ablation, electron irradiation, gamma irradiation, microwave processing, chemical reduction, photochemical methods, and biological synthetic methods. The most common method for the preparation of silver NPs is chemical reduction that produce stable, colloidal dispersions in water or organic solvents^15^. In Dentistry, two mechanisms are applied for bacterial reduction: a) combining dental materials with NPs; and b) coating surfaces with NPs to prevent microbial adhesion^13^. In this study, we used the second mechanism and for the first time silver NPs were applied with etch-and-rinse and self-etch adhesive systems as pretreatment. The rationale was to achieve benefits of antibacterial properties and perhaps positive effects on bond strength.

Although discoloration and color changing to a tone of gray are common problems in all materials containing silver, especially composite resins, in the present study we did not see silver spots by visual eye check on any tooth, but we did not check it with the microscope either. It is showed in the previous study that using low concentrations of metal NPs can prevent severe discoloration of composite resins. Therefore, according to previous studies we chose concentrations of 1% silver NPs to impart the less detrimental effect of these NPs on the color of composite resins^16^.

From this study it can be concluded that the bonding effectiveness of Clearfil SE Bond can be improved by selectively use of silver NPs on the walls of the cavity. An antibacterial bonding agent at the tooth-restoration interface is very important because usually there are bacteria in the prepared tooth cavity. In previous studies, primer and adhesives that contain silver NPs could kill the residual bacteria^8,18^. Bacteria at the interface of the tooth-restoration margins could harm the dental pulp and also affect bond strength. The primer could be an important vehicle to deliver antimicrobial agents such as silver NPs to kill bacteria in the tooth cavity because it has direct contact with dentin^10,30^. Silver NPs has a strong antibacterial activity, low cytoxicity and acceptable biocompatibility with human cells, and a long-term antibacterial effect by means of sustained silver ion release^6^. The development of new self-etch adhesives offers some promising opportunities. Unlike with etch and rinse adhesives, not all hydroxyapatites are removed from the hybrid layer in dentin as the demineralization by mild self-etch adhesives occurs. Depth and extent of demineralization by mild self-etch adhesives is limited compared to etch-and-rinse^20,28^. Researches have shown that functional monomers in self-etch adhesives can chemically interact with hydroxyapatite within a clinically acceptable time^7^, and this chemical interaction has been hypothesized to be improved by possible infiltration of silver NPs into dentinal tubules and perhaps it could provide better resistance against degradation by prevention of micro- and nanoleakage.

The present study demonstrated that bond strength of etch-and-rinse adhesive, (Adper Single Bond) was higher than self-etch (Clearfil SE Bond) adhesive, using silver NPs, under SBS test. Therefore, it seems that the effect of etching in the etch-and-rinse adhesive on cavity surfaces has an important role in producing high bond strength. In a previous study, 250 ppm and 500 ppm of silver NPs with a size smaller than 5 nm in combination with nanosized silica particles were added to self mixed experimental composite adhesives. They found that silver NPs did not significantly affect SBS among adhesives^2^. However, we added silver NPs with different protocols. Bonding agents seemed to have infiltrated and wetted the dentin surface well, after silver NPs application. It might perhaps change the etching pattern of phosphoric acid and form the long resin tags from well-filled dentinal tubules. They also found that an experimental composite adhesive containing silica nanofillers and silver NPs can help prevent enamel demineralization around bracket surfaces without compromising physical properties^2^. Yoshida, et al.^29^ (1999) demonstrated that a resin composite incorporated with silver NPs had a long-term effect against *Streptococcus mutans*. In addition, they showed that a resin composite that has fillers containing Ag ions had antibacterial effects on oral streptococci with no adverse effect on adhesive mechanical properties, allowing thus their use in restorative treatments^29^.

The present study measured dentin bond strength after one week of water-aging. More studies should be conducted to investigate the long-term bond durability of adhesive systems after long-term water-aging to investigate the effects of silver ion release and its effects on dental hard tissue remineralization, antibacterial characteristics, and the durability of the resin-dentin bond. In our *in vitro* study, we did not investigate the release of silver NPs into oral cavity and saliva, but these studies should be considered. It seems good to emphasize that more researches are needed to confirm these results, in which other types of adhesives and other methods that can produce similar conditions of degradation of the adhesive interface in the oral cavity should be performed. Furthermore, the results of this study were developed *in vitro*, therefore, clinical investigations are required to establish these findings and provide clinical recommendations.

## Conclusion

Considering the positive antibacterial effects of silver NPs, using them is recommended in restorative Dentistry. It seems that silver NPs could have positive effects on bond strength of both etch-and-rinse and self-etch adhesive systems. The best results of silver NPs have been achieved with Adper Single Bond and before acid etching.
